# Study on Antibacterial Activity of Water Extract of *Galla chinensis* Against Carbapenemase‐Producing *Enterobacteriaceae*

**DOI:** 10.1002/mbo3.70041

**Published:** 2025-07-28

**Authors:** MinHui Miao, Jiayao Lin, Jie Zhu, Hong Du, Di Mei, AnQi Gu, BeiNa Hu, HaiQiang Jiang

**Affiliations:** ^1^ Jiangyin Hospital of Traditional Chinese Medicine Jiangyin Hospital Affiliated to Nanjing University of Chinese Medicine Jiangyin China; ^2^ Department of Clinical Laboratory The Second Affiliated Hospital of Soochow University Suzhou China

**Keywords:** antimicrobial resistance, carbapenemase‐producing *Enterobacteriaceae*, Galla chinensis, gene expression, transcriptome analysis

## Abstract

Carbapenemase‐producing *Enterobacteriaceae* (CPE) represents a significant global public health concern, largely driven by factors including misuse of antibiotics and their inappropriate use in livestock. This study systematically evaluated the minimum inhibitory concentration (MIC) of *Galla chinensis* (GC) water extract against a range of CPE strains, aiming to assess its potential therapeutic application and efficacy against multidrug‐resistant bacteria (MDR). MIC values for carbapenemase with different plasmids spanned between 3.9 and 500 mg/mL, while readings of 62.55–500 mg/L demonstrated no colonies. The growth curve analysis demonstrated that the GC extract significantly inhibited bacterial growth at both 1/4 and 1/2 MIC doses, completely inhibiting bacterial growth at 1 MIC (*p* < 0.01). Furthermore, the effect of 1/4 MIC was also statistically different; however, it may not provide a meaningful inhibitory effect. qRT‐PCR analysis revealed an increase in the expression of carbapenemase genes, including *pkPC2_InFII*, *pNDM5_IncX3*, *pIMP4_IncN*, and *pOXA48_IncX3* subsequent to GC extract treatment at 1/2 MIC. Transcriptomic analysis further revealed differential gene expression associated with bacterial resistance mechanisms. Consequently, the GC extract demonstrated pronounced antibacterial efficacy against CPE, exhibiting a significant ability to inhibit their growth and minimize the activity of vital resistance genes.

## Graphical abstract

*Galla chinensis* is an eco‐friendly and effective alternative to traditional antibiotics for combating multidrug resistance.

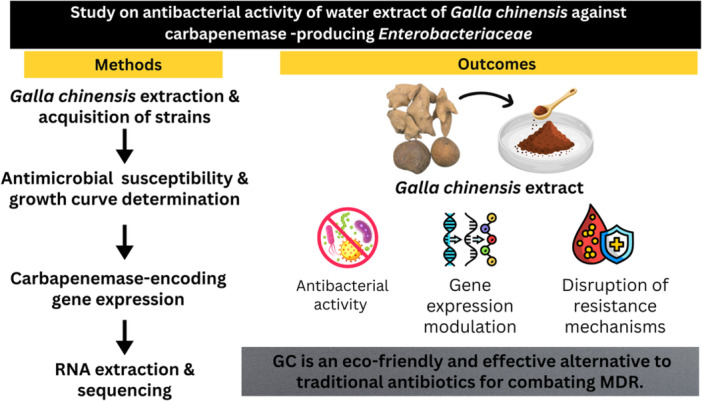

## 1 Introduction

Antibiotic resistance represents a global health concern, involving the transmission of both bacteria and genetic material among humans, animals, and the surrounding environment (Larsson and Flach 2022). The emergence of antibiotic resistance in bacterial populations accounts for numerous deaths annually, amounting to hundreds of thousands. The most debated issue is the increase in the number of bacteria becoming resistant to widely used antibiotics (Urban‐Chmiel et al. 2022). Antibiotic resistance may emerge as a result of mutations occurring within the pre‐existing genomic of a bacterium, as well as from the acquisition of foreign DNA (Schulz et al. 2017). Consequently, many developing countries face increased susceptibility to antibiotic resistance, driven by factors including unregulated antibiotic usage, hospital overcrowding, poor hygiene, a higher infection burden, and restricted access to costly antibiotics (Laxminarayan et al. 2020).

Within *Enterobacteriaceae*, antibiotic resistance is high and increasing, with the majority of these bacteria reside in the human gut. A number of infections of *Enterobacteriaceae* have traditionally been treated with the beta (β) lactam class of antibiotics, such as penicillins, cephalosporins, monobactams, and carbapenems. (Hsu et al. 2016; Kong et al. 2010). Antibiotic‐resistant genes, including transposons and plasmids, are transferred through mobile genetic elements (MGEs), contributing to the worldwide emergence of carbapenemase‐producing *Enterobacteriaceae* (CPE) (Logan and Weinstein 2017). Moreover, pathogens with elevated mortality rates within the CPE spectrum, including *Escherichia coli* and *Klebsiella pneumoniae*, represent a serious global threat to public health (Mathers et al. 2020). Consequently, many scholars are increasingly interested in exploring strategies to address MDR, leading to a growing interest traditional Chinese medicine (TCM). (Li et al. 2022). This interest is supported by a historical legacy and a substantial body of evidence regarding the control of infectious diseases, making TCM a viable option. The antimicrobial activity of TCM and its constituent compounds involves multiple mechanisms, targeting and interacting with a refined and elaborated process (Wu et al. 2019).

Gall formation induced by *Melaphis chinensis* (Bell Baker) via stimulation of plant leaves is represented by *Galla chinensis* (GC), a substance historically recognized for its medicinal properties according to TCM throughout the East Asia (Ren et al. 2021). Its initial documentation as a therapeutic agent can be traced back to the ancient Chinese medical text “Bencao Shiyi,” authored during the Tang Dynasty. Currently, GC is refined through the elimination of impurities and nontherapeutic components, and it is commonly prepared for oral consumption or as a powder to manage conditions such as diarrhea, hemorrhage, folliculitis, fistula, chronic cough, mucus, and oral ulcers (Liu et al. 2024; Nguyen et al. 2024; Yan et al. 2024). A considerable amount of literature has investigated the anti‐pathogenic microorganism effect of GC. Specifically, researchers have examined the effects of GC extracts on inhibition of various types of infections, including *Vibrio harveyi* (Pan and Yan 2020), *Vibrio parahaemolyticus* (Wu et al. 2016), and the inhibition of HCV Core 1b and NS5A proteins (Kwon et al. 2019).

Extracts from GC, encompassing gallic acid, gallotannins, and hydrolyzable tannins, have demonstrated notable antibacterial properties (Ren et al. 2021; Tian et al. 2009). Furthermore, safety assessments have indicated that GC extracts exhibit an almost negligible level of toxicity when administered at low concentrations, irrespective of whether considered in acute or chronic settings (Zhang et al. 2019). Prior research addressing the effects of the GC or its extract on the *Enterobacteriaceae* family, including *E. coli* and *Enterobacter cloacae*, has been documented (Manilal et al. 2023). However, limited evidence exists regarding the definitive antibacterial performance of GC extract in relation to CPE. Therefore, this paper primarily addresses the following questions: (1) the evaluation of the antibacterial efficacy of GC aqueous extract in relation to CPE; (2) the assessment of changes in the gene expression profile of CPE's carbapenemase genes following GC aqueous extract treatment; and (3) the identification of the transcriptomic response of CPE to the GC aqueous extract.

## 2 Materials and Methods

### 2.1 Preparation of Aqueous Extract of GC

GC was procured from Beijing Tongrentang pharmacy, a recognized establishment specializing in TCM practices. Following a 2‐h immersion period at 25°C in a 1:10 ratio of distilled water, the GC underwent a decoction involving elevation of the mixture's temperature to 100°C for 1 min, followed by gentle simmering for 30 min. The resulting decoction was then filtered using six layers of gauze. The decoction process was concluded with re‐immersing the filter residue in distilled water at a ratio of 1:10. Utilizing a Rotavapor (Shanghai Yarong Biochemical Instrument Factory), the blended decoctions were concentrated to a crude drug density of 1 g/mL. Subsequently, these preparations were subjected to autoclave sterilization at a temperature of 121°C and stored at 4°C (Shanghai Boxun Medical Biological Instrument Co. Ltd). The concentration of polyphenolic compounds in the GC extract was estimated using UV‐visible spectrophotometry at 270 nm, with gallic acid as the calibration standard.

### 2.2 Bacterial Strains

The bacterial strains utilized in the present investigation originated from the human sources were obtained from the Department of Laboratory Medicine at Soochow University, Second Affiliated Hospital in Suzhou, Jiangsu, China. These particular strains, utilized as conjugation transfer strains, were clinically derived and possessed carbapenemase resistance genes encoded within plasmids, functioning as donor strains. The recipient strains were identified as *E. coli* J53 or E600, well‐documented in the literature for their role in bacterial conjugation and antibiotic resistance transfer. The plasmids employed in this study encoded a variety of resistance genes, including *pNDM1*_*IncC*, *pNDM5*_*IncX3*, *pKPC2*_*IncFII*, *pIMP4*_*IncN*, *pIMP4*_*IncU*, and *pOXA48*_*IncX3* each of which has been previously associated with significant clinical implications regarding antimicrobial resistance.

### 2.3 Testing Planktonic Antimicrobial Susceptibility

Strains with 1 × 10^6^ CFU/mL were tested using broth microdilution assays to determine minimum inhibitory concentration (MIC) in nutrient‐rich Luria‐Bertani (LB) medium. A 96‐well microtiter plate was prepared, with each well containing 100 µL of sterile LB medium to ensure a controlled environment. Following the addition of 200 µL of GC water extract at a concentration of 1000 mg/mL to the first well, a serial dilution was performed to achieve a range of concentrations. Subsequently, 10 µL of a standardized bacterial suspension (1 × 10^7^ CFU/mL) was added to each well, and the plates were incubated for 24 h to promote bacterial growth. The GC water extracts, diluted to concentrations ranging from 3.9 to 500 mg/mL, were utilized in triplicate assay to ensure experimental reliability and reproducibility. MIC values were determined based on the absence of bacterial growth.

### 2.4 Growth Curve Determination

To assess the effect of GC water extract on bacterial growth, strains exhibiting exponential growth—characterized by an optical density at 600 between 0.5 and 0.7—were collected, centrifuged at 8500 × *g* for 10 min, and resuspended in fresh LB medium. The extract was added at concentrations of 1/4, 1/2, and 1 MIC. Two hundred microliters of each sample was then dispensed into 96‐well plate, which was incubated at 35°C. Bacterial growth was monitored hourly basis at 600 nm using a Rayto RT‐6000 Microtiter Plate Reader (Shanghai Enzyme‐Linked Biotechnology Co. Ltd., China). Growth curves were generated throughout the 24‐h using GraphPad Prism version 10.1, and statistical significance *p* < 0.05 was determined via a non‐parametric Kruskal–Wallis test.

### 2.5 Determination of Carbapenemase‐Encoding Gene Expression

Quantitative real‐time polymerase chain reaction (qRT‐PCR) was employed to assess the expression levels of several genes, including *pkPC2*_*InFII*, *pNDM5*_*IncX3*, *pIMP4*_*IncN*, and *pOXA48*_*IncX3*, within the bacterial strains. Following an overnight incubation at 37°C with continuous shaking (250 rpm) in 1 mL LB broth, Strains were diluted 1:100 into 10 mL of the fresh LB medium and allowed to reach an optical density of 600 nm = 0.5. At this point, a specific concentration (1/2 MIC) of GC water extract was added into the cultures. Total RNA was extracted using the RNeasy Kit (Qiagen, Germany), following manufacture's instructions. To ensure a comparative analysis, a control group that was entirely free from the GC extract was maintained and monitored throughout the duration of the study. The measurement of RNA concentration was conducted using NanoDrop 2000 (Thermo Scientific, USA). c(DNA) synthesis was performed using PrimeScript RT Master Mix (Takara, Japan) with Oligo dT Primer and Random 6 mers. The reaction mixture was incubated for 15 min at 85°C and subsequently inactivated for 5 s at 85°C. Before being used, the generated cDNA was kept at −80°C.

A Roche 96 Instrument (Roche Diagnostics GmbH, Switzerland) was utilized for real‐time PCR using the Thunderbird SYBR qPCR Mix (TOYOBO, Japan). Following a 40 cycles protocol including denaturation for 15 s at a temperature of 95°C, subsequent annealing for 30 s at 60°C, and extension for 20 s at 72°C, a comprehensive melting curve analysis was performed. The initial denaturation phase consisted of 1 min at 95°C. Primers were manufactured by Jinweizhi Biotechnology, designed with National Center for Biotechnology Information (NCBI) resources. The 2^−ΔΔ*CT*^ method (Livak and Schmittgen 2001) was used to determine relative expression, with 16S rRNA serving as the reference gene. Each experimental procedure was three times to ensure reproducibility and reliability.

### 2.6 RNA Extraction and Transcriptome Sequencing

In the transcriptomic control group, no GC extract was added, while all other culture conditions were identical to those used for the experimental groups. Total RNA was isolated from both the control and GC extract‐treated groups (at 1, 1/2, and 1/4 MIC) using the MasterPure RNA purification kit (Epicenter Technologies, Madison, WI, USA), followed by DNase I purification (Ambion). RNA quality and purity were assessed via an Agilent 2100 bioanalyzer (Agilent Technologies, CA, USA), targeting RNA integrity numbers (RIN) of 9.0 or higher. Ribosomal RNA was removed using a Ribo‐Zero rRNA Removal Kit designed for gram‐positive bacteria (Epicenter). Illumina TruSeq RNA sample preparation kit (Majorbio Biotechnology Research in Shanghai, China) was utilized to generate cDNA libraries, which were subsequently sequenced on a HiSeq. 4000 platform (2 × 150 bp read length). Following the alignment of the CRE genome to the sequencing data, differential gene expression analysis was conducted using the edgeR package. Also, the RNA‐seq reads were normalized before analysis using the FPKM (Fragments Per Kilobase of transcript per Million mapped reads) method, which accounts for both sequencing depth and gene length.

### 2.7 Statistical Analysis

RNA libraries were generated from enriched mRNAs using the Illumina TruSeq RNA sample preparation kit. Majorbio Biotechnology Research (Shanghai, China) conducted RNA sequencing (RNA‐Seq) on a HiSeq. 4000 platform with a read length of 2 × 150 bp. Sequencing reads were aligned to the organism's genomic sequence using Bowtie on a Galaxy server. EdgeR was used to analyze the data, identifying variations in fold changes and statistically significant differences in gene expression between the control and treatment cohorts (http://www.bioconductor.org/packages/2.12/bioc/html/edgeR.html). Statistically significant differential expression was defined by a fold‐change exceeding two, alongside a false discovery rate (FDR) that remained below 0.05, ensuring the reliability of the results. Gene ontology (GO) terms were utilized for pathway enrichment analysis, leveraging the Blast2GO software tool. Fisher's exact tests were systematically employed to compare enrichment levels against the background GO terms. The FDR criterion of less than 0.05, as articulated by Lewin and Grieve (2006), was implemented to account for multiple hypothesis testing, thereby enhancing the validity of the statistical conclusions.

### 2.8 Data Analysis

Statistical analysis was conducted using SPSS version 16.0 (SPSS Inc., Chicago). The normality of the collected data were assessed using Shapiro–Wilk tests. One‐way analysis of variance (ANOVA) and independent *t*‐tests were applied as required, with significance thresholds set at *p* < 0.05.

## 3 Results

### 3.1 Antibacterial Activity of GC Extracts Against CPE

GC extract underwent a systematic two‐fold dilution process, ranging from 3.9 to 500 mg/mL, to determine the MIC values and assess antibacterial efficacy against clinically significant CPE. These results, depicted in Table 1, provide a comprehensive overview of the MIC values observed for various transconjugant strains, contributing to a broader understanding of the antibacterial properties of GC extract. When analyzing the Clinical Reference Endpoint, the GC extract exhibited a MIC range of 62.5–125 mg/mL for *E. coli*. Notably, the majority of transconjugant strains consistently exhibited MIC values of 62.5 mg/mL, including various strains of *E. coli* E600 carrying plasmids such as *pNDM5*_*IncX3*, *pKPC2*_*IncFⅡ*, *pIMP4*_*IncN*, *pOXA48*_*IncX3*, and the *E. coli* J53 strain with the *pNDM1*_*IncC* plasmid. In contrast, specific *E. coli* E600 strains carrying the *pIMP4*_*IncU* plasmid represented a significantly elevated MIC value of 125 mg/mL, highlighting a distinct resistance profile.

**Table 1:** Overview of the MIC of the GC extract when subjected to carbapenemase‐producing *Enterobacteriaceae*.

			(mg/mL)							
Strains	Plasmid	Carbapenemase	3.9	7.8	15.6	31.3	62.5	125	250	500
E600	IncX3	NDM‐5	+	+	+	+	—	—	—	—
E600	IncFII	KPC‐2	+	+	+	+	—	—	—	—
E600	IncN	IMP‐4	+	+	+	+	—	—	—	—
E600	IncU	IMP‐4	+	+	+	+	+	—	—	—
E600	IncX3	OXA‐48	+	+	+	+	—	—	—	—
J53	IncC	NDM‐1	+	+	+	+	—	—	—	—

*Note:* The symbol “+” denotes the presence of existing colonies, while the symbol, “—” indicates an absence of any colony growth.

### 3.2 Growth Curves of Bacteria Under GC Extract Pressure

**Figure 1:**
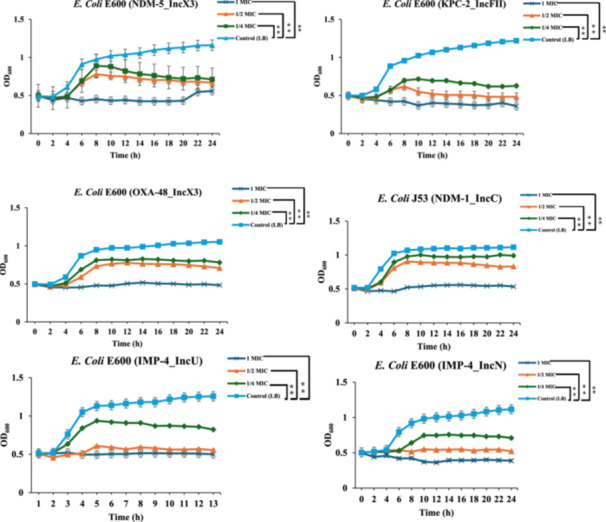
Determination of the growth kinetics of CPE‐conjugated strains subjected to varying concentrations of GC water extracts at OD_600_. The bacterial cultures were cultivated in LB broth supplemented with differing concentrations of GC extracts (1, 1/2, and ¼ MIC), with the LB bacterial suspension devoid of GC extract serving as the negative control group. The results were statistically analyzed using the Kruskal–Wallis test, represented the means of three independent experiments with **p < 0.01. The error bars illustrated the standard deviations calculated from three replicates and the symbol “**” indicates highly significant difference compared to control.

Figure 1 presents the growth curves associated with CPE subjected to different concentrations of GC water extracts, a vital component of this analysis. At concentrations equivalent to (1/4 MIC) and (1/2 MIC), the GC water extract significantly inhibited bacterial growth, effectively delaying the exponential growth phase in bacterial populations. The application of GC extract at 1 MIC unequivocally inhibited the growth of CPE, illustrating its profound impact on bacterial resistance mechanisms. As depicted in the figure, notable differences in the growth rates can be observed among the various strains of CPE that have undergone conjugation, highlighting the influence of genetic factors on their growth. Specifically, strains harboring the plasmid *pNDM5*, such as *E. coli* E600, exhibited a markedly prolonged transition into the logarithmic growth phase, approximately 2 h longer than the control group that did not receive any treatment. Furthermore, the other strains such as *E. coli* E600 (*pKPC2* from the *IncFII* group), and *E. coli* E600 with the plasmid *pOXA48*_*IncX3* group also demonstrated this same delayed growth characteristic. These findings underscore the importance of considering genetic factors, like the presence of specific plasmids, when evaluating the efficacy of GC extracts against CPE.

Analysis of the bacterial strain *E. coli* J53 (*pNDM1*_*IncC*) revealed a distinct growth cycle comprising a delayed phase, logarithmic phase, and stable phase mirroring observations within the control group, 1/2 MIC group, and the 1/4MIC group. However, comparative analysis with other bacterial strains demonstrated a less pronounced reduction in overall bacterial counts within the 1/4MIC group and 1/2 MIC group. Specifically, in the case of *E. coli* E600 (*pIMP4*_*IncU*) and *E. coli* E600 (*pOXA48*_*IncX3*), application of the 1/2 MIC GC extract resulted in near‐complete inhibition of growth, characterized by the absence of a clearly defined logarithmic growth phase. These findings collectively suggest that GC extracts exert varying degrees of inhibitory effects on the growth of these conjugated resistance strains of CPE, highlighting their potential role in managing antibiotic resistance within bacterial populations.

### 3.3 Expression of Carbapenemase‐Encoding Genes in Different Plasmids After GC Extract Treatment

**Figure 2:**
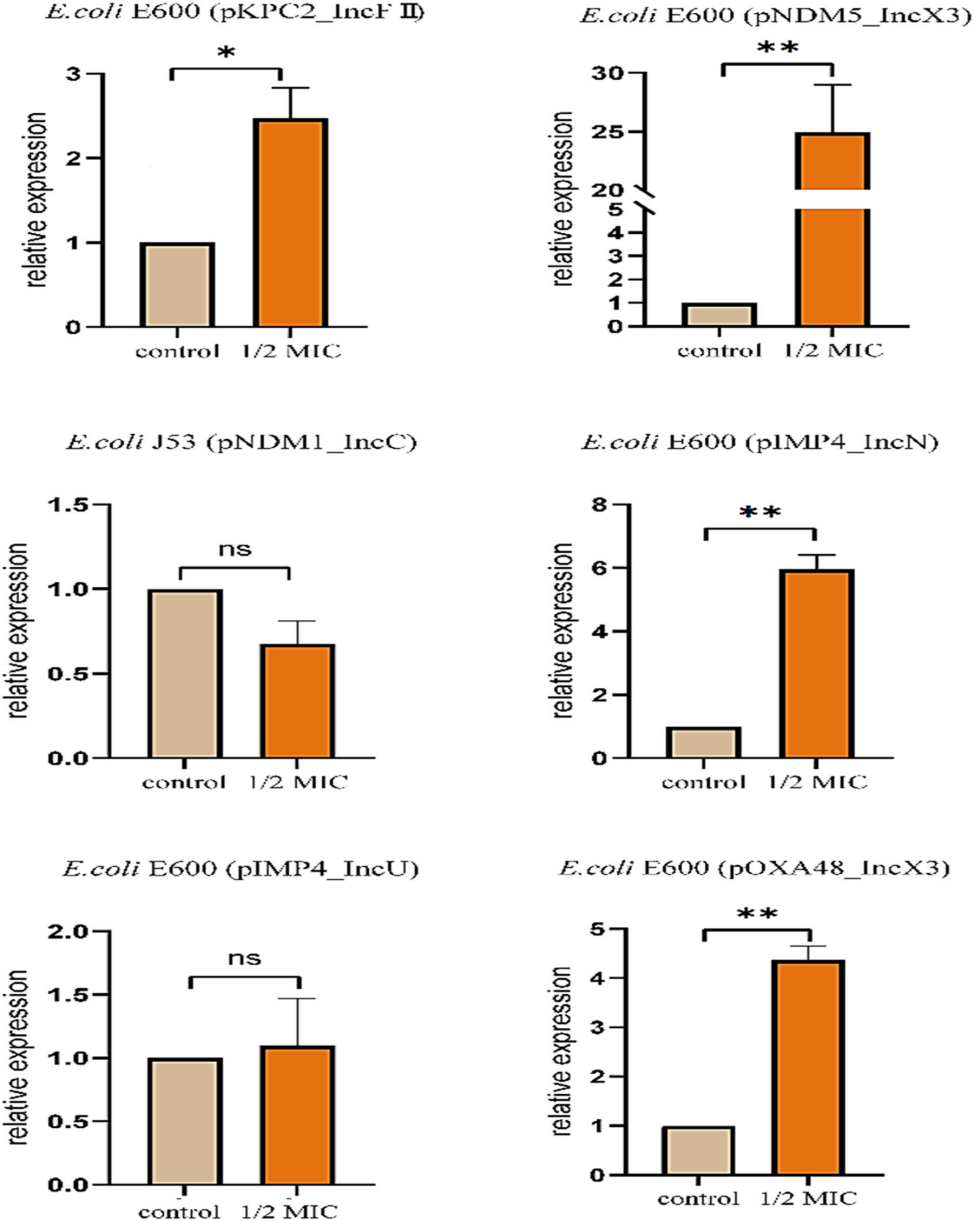
The utilization of GC extract across a range of different concentrations has been investigated. The abbreviation “ns” denotes nonsignificant results, the symbol “*” signifies a statistical difference compared to control, and the symbol “**” indicates highly significant difference compared to control.

Following treatment with 1/2 MIC of GC extract for 120 min, a comprehensive examination revealed alterations in the expression levels of genes encoding carbapenemase, located on distinct plasmids, in four selected bacterial strains. As illustrated in Figure 2, the application of GC extract resulted in a significant upregulation of these genes. Specifically, the expression of *blaKPC‐2* (present on the IncFⅡ plasmid), *blaNDM‐5* (present on the *IncX3* plasmid), *blaIMP‐4* (found on the *IncN* plasmid), and *blaOXA‐48* (on the *IncX3* plasmid) was markedly increase. The expression of *blaNDM‐5* on the *IncX3* plasmid exhibited the greatest upregulation, increasing by a factor of 25, whereas the expression of *blaKPC‐2* on the *IncFII* plasmid displayed a comparatively modest increase of 2.5‐fold. In contrast to these findings, no statistically significant changes were observed among the analyzed parameters. In contrast to these findings, no statistically significant changes were detected in the expression levels of *blaNDM‐*1 gene (present on the IncC plasmid), regardless of the GC extract treatment. The RNA expression level of the *blaIMP‐4* gene demonstrated substantial variability, exhibiting a pronounced sixfold upregulation of sixfold on the IncN plasmid while maintaining a stable expression level on the IncU plasmid.

### 3.4 Transcriptome Analysis of *E. coli* E600 (pNDM5_IncX3)

**Figure 3:**
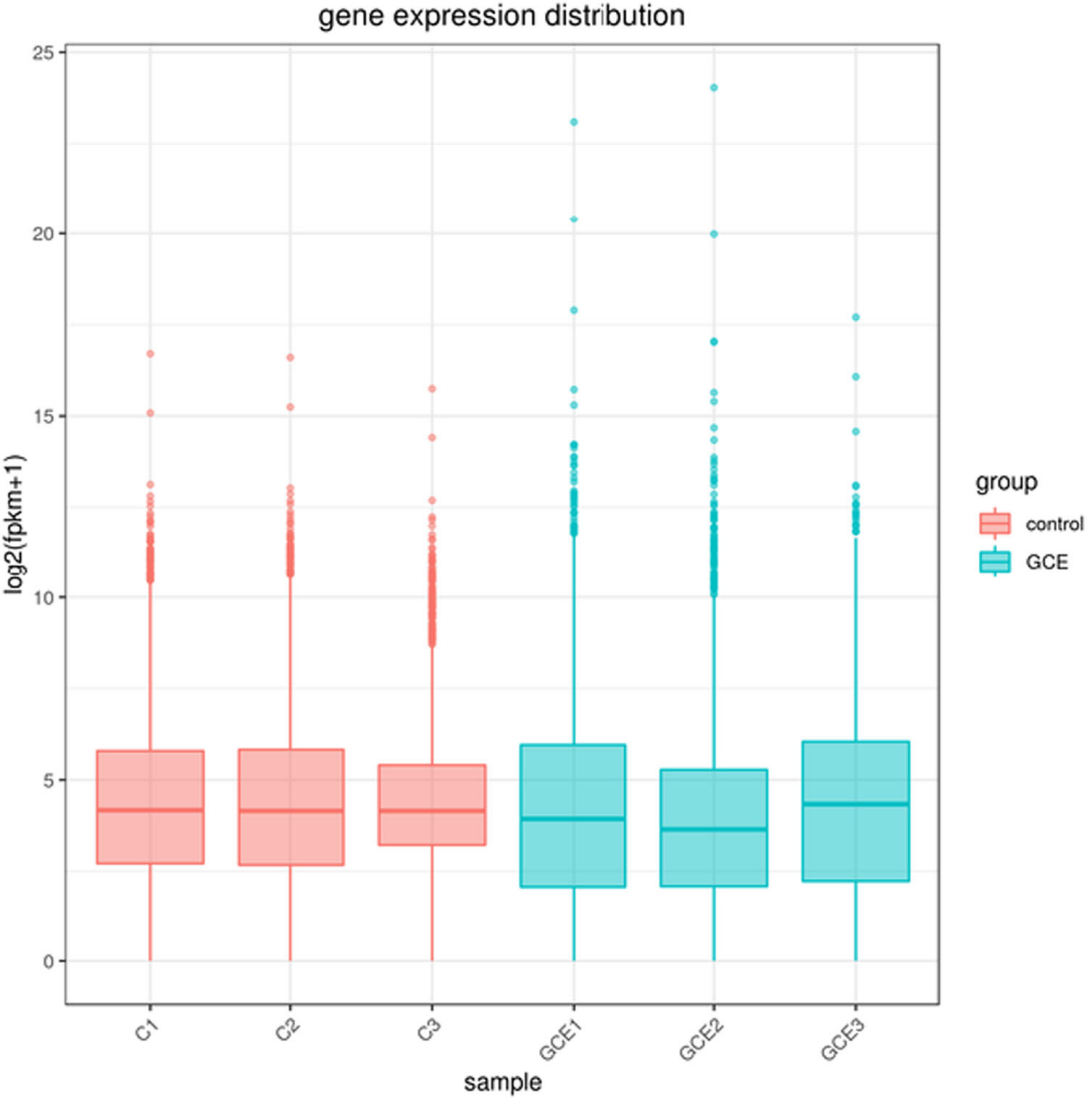
The boxplot shows the analysis of the varying distributions of gene expression levels across six samples reveals intricate patterns underlying mechanisms of gene regulation and their implications.

**Figure 4:**
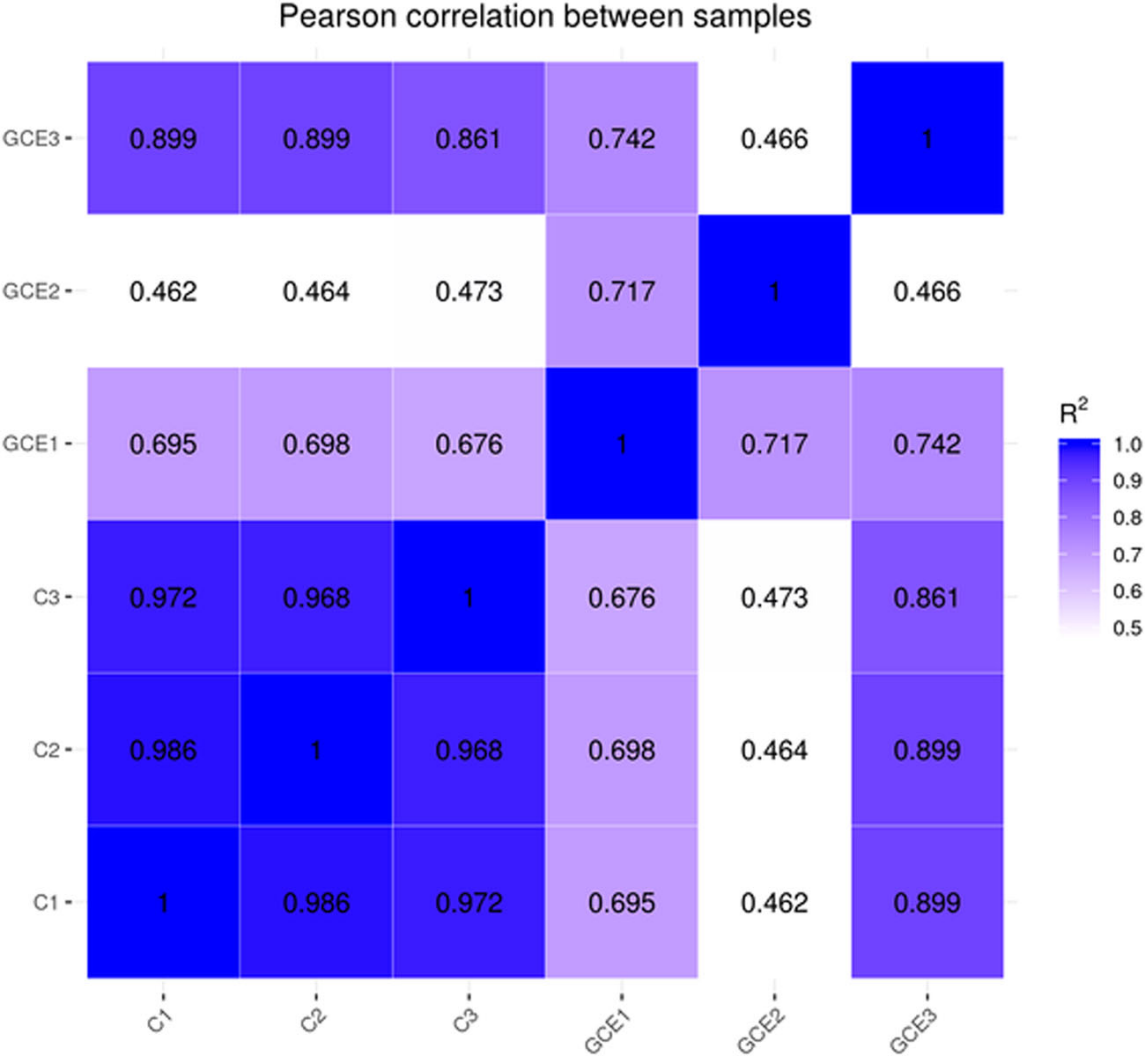
Gene expression correlation diagram. The horizontal axis, as well as the vertical axis, represents the squares of the correlation coefficients for each individual sample analyzed within the scope of the study, providing a comprehensive relationship between the various gene expression levels.

**Figure 5:**
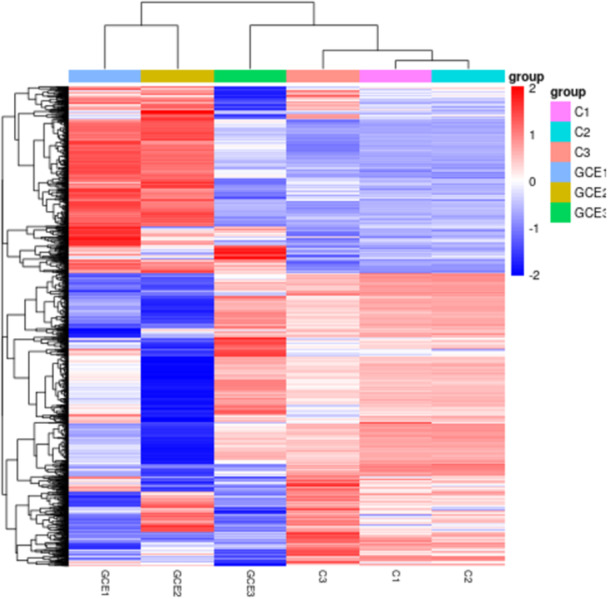
Differential gene clustering heat map. The horizontal axis represents the nomenclature of the samples utilized in the analysis, while the vertical axis illustrates the normalized values corresponding to the expression levels of the differentially expressed genes, specifically measured in terms of Fragments Per Kilobase Million (FPKM).

A thorough analysis of differential gene expression following the experimental treatment showed significant modifications in biological pathways associated with mechanisms of bacterial resistance, including efflux pumps, membrane transport processes, and various metabolic pathways. As demonstrated in Figure 3, the gene expression levels within the control group (samples C1, C2, and C3) exhibited a remarkable degree of consistency, suggesting minimal heterogeneity among these samples. In contrast, the expression levels of the genes GCE1, GCE2, and GCE3 revealed significantly greater variability compared to the control group, with a noteworthy prominence in the expression of GCE2. GO and pathway enrichment analyses depicted significant disruptions in critical biological processes and molecular functions, thereby elucidating the multifaceted antibacterial properties of the GC extract, which are of considerable interest within microbiological research. Notably, the expression of genes encoding for carbapenemase, frequently located on plasmids, was observed following GC extract application, indicating a potential response mechanism of the bacteria to this therapeutic intervention. Furthermore, analysis of Figure 4 revealed lower correlations between the control group and the group treated with GC extract, suggesting a significant specificity associated with GC extract effects on gene expression. This particular observation strongly suggests that the treatment administered through GC extract has led to noteworthy alterations in the gene expression profiles. The trend of high correlation within the control group, as represented in Figure 5, confirms the reliability of the experimental setup and shows that the gene expression profiles are consistent under control conditions. On the other hand, the lower correlations and increased variability in the control and GC extract groups reflect its impact on gene expression. The findings demonstrate that the treatment leads to profound changes in the gene expression profile, supported by correlations within the GC extract group. Consequently, the observed variability suggests relatively heterogeneous responses in gene expression. Differential expression analysis also revealed a considerable quantity of regulated genes in response to treatment with GC extract. Among these genes, several implicated in membrane transport, amino acid biosynthesis, and oxidative phosphorylation were observed to be upregulated, whereas others linked to ribosomal function and primary metabolism exhibited downregulation. GO enrichment analysis demonstrated that differentially expressed genes were particularly enriched in biological processes related to transmembrane transport, ion homeostasis, and protein synthesis. These results imply that exposure to GC extract initiates extensive transcriptional reprogramming, potentially associated with bacterial stress adaptation and antimicrobial defense mechanisms. This variability may arise from underlying biological sources or alterations in treatment pathways and mechanisms. Further investigation into the molecular mechanisms and biological significance of these observed changes in gene expression is needed.

## 4 Discussion

The increasing prevalence of MDR strains, driven by utilization of antibiotics, represents a significant and concerning challenge to current therapies. Therefore, carbapenem antibiotics, often considered a last‐line defense against bacterial infections, are increasingly utilized. However, certain bacterial strains, specifically CPE, have developed the ability to produce enzymes capable of degrading carbapenem antibiotics. Therefore, the need of the hour to research into alternative therapeutic strategies and the evaluation of products which are derived naturally, such as GC is urgently needed. A promising antibacterial compound in GC, is primarily composed of tannin, synthesized through the polymerization of 7–9 molecules of gallic acid along with one molecule of glucose. The concentration of this tannin ranges from approximately 60%–70% and can even reach up to 80%. Its applications span across the medicine, chemical industry, and the food sector, thereby establishing it as a focal point of inquiry within the research community (Xie et al. 2019).

GC extract revealed its efficacy in inhibiting various CPE strains, each possessing a specific plasmid, with MIC values ranging from 3.9 to 500 mg/mL (Table 1). At concentrations below the threshold of 3.9–31.3 mg/mL, the FC extract failed to have an observable antimicrobial effect, allowing the bacteria to remain unaffected. This key observation is in line with the dose–response relationships, which states that, when the concentrations of an antimicrobial agent are inadequate, the resultant effects are nonsignificant (Hancock 1998). These observations align with previous research indicating that the MIC of GC ethanol extract is significantly enhanced at higher concentrations, exhibiting notable antibacterial properties against *Enterococcus faecalis* and other species (Li et al. 1993). Furthermore, our findings are also aligning with Friedman et al. (2004), who reported increased antimicrobial activity in GC due to the presence of bioactive compounds, notably gallic acid and gallotannins. This effect may be attributed to the diverse natural compounds found within GC. Related research has also highlighted the roles of ellagitannins in fruits and nuts and gallotannin in *Terminalia chebula* (Kim et al. 2013; Lipińska et al. 2014), alongside baicalein from the root of *Scutellaria baicalensis* (Chan et al. 2011).

Assessment of the effect of GC extract at concentrations of 1, 1/2, and 1/4 MIC on the growth kinetics of CPE‐conjugated strains, as depicted in Figure 1, revealed bactericidal properties at each concentration. Various experiments regarding plant extracts have demonstrated the same results, indicating that these extracts mitigate antibiotic resistance various mechanisms, including targeting bacterial cellular envelopes, enzymatic activities, and metabolic pathways (Cushnie and Lamb 2011; Friedman 2015). Specifically, the mechanism action of GC extract may be attributed disruption of the bacterial membrane. For instance, a study conducted by (Friedman et al. 2004) demonstrated that exposure of the *Bacillus cereus* to carvacrol resulted in significant alterations within the cellular environment, including ATP depletion and a change in membrane potential, subsequently increasing the permeability of the cytoplasmic membrane. The disruption of the ion gradient that is vital for maintaining cellular homeostasis severely impairs essential metabolic processes within the cell, ultimately restricting in a process that leads to cell death, thus demonstrating the profound impact of carvacrol on the viability of *B. cereus*. It is worth mentioning that in Figure 1 each MIC of GC extract significantly (*p* < 0.05) reduced the growth of the bacteria, but the lower concentration (MIC at 1/4) was performing less than the other levels of GC extract (1 and 1/2 MIC). This response is attributed to the lower number of bioactive compounds at 1/4 MIC. Our results match the findings of (Kowalska‐Krochmal and Dudek‐Wicher 2021), in which they highlighted that low MIC levels can trigger resistance mechanisms, and highlight the consistent efficacy of higher concentrations aligned with clinical therapeutic practices.

The application of GC extract upregulated carbapenemase‐encoding genes (Figure 2) (*pkPC2*_*InFII*, *pNDM5*_*IncX3*, *pIMP4*_*IncN*, and *pOXA48*_*IncX3*)) known for carbapenem resistance and frequently associated with MDR strains—further highlighting the complex interaction between natural products and bacterial resistance mechanisms (Meletis 2016). There are number of reasons behind this upregulation, but the expression in reaction to GC extract application might be due to the activation of resistance mechanism, which is induced by the phytochemicals present in GC extract (Gashaw et al. 2024). Another possible reason for this phenomenon may also be linked to the diverse nature of compounds acting as signaling molecules that trigger gene expression (Alav et al. 2024).

Transcriptome analysis provided further insights into the molecular alterations induced by GC extract in CPE. Previous literature highlighted the disruption in normal metabolic pathways can significantly enhance the effect of antimicrobial therapies by compromising the ability of bacteria to survive under stress (Gashaw et al. 2024). The results presented in Figures 1 and 2 suggest that GC extract may influence metabolic processes, potentially leading to inhibition of certain pathways. However, further investigation is needed to confirm the extent and mechanism of this effect (Sb et al. 2023). In conjunction with its impact on metabolic and stress response mechanisms, the effect of GC extract on virulence factors is paramount for its antibacterial efficacy (Tjaden et al. 2002). Furthermore, previous research has demonstrated that GC exhibits low toxicity and minimal side effects, thereby supporting its therapeutic potential. In a study conducted by Xiang et al. (2015), they demonstrated no adverse effects were reported on rats. Further research on GC extract is essential for conclusive findings and investigating GC's bioactive components may lead to innovative therapies for MDR, yielding favorable results.

## 5 Conclusion

This study extends our knowledge to determine the effect of antibacterial potential of GC extract on CPE, by revealing several mechanisms of action. Although the current study is based on the extract of GC on CPE, but the findings suggest that GC extract not only have antimicrobial properties but also disrupts normal metabolic process, virulence pathways, and stress response highlighting a broad‐spectrum activity that could help in the reduction of MDR. In the future, more research is needed on specific compounds of GC to have a whole picture in evaluation and safety of GC extract in clinical settings.

## Author Contributions

**MinHui Miao:** conceptualization, writing – original draft; methodology. **Jiayao Lin:** conceptualization, writing – original draft, methodology. **Jie Zhu:** conceptualization, methodology, software. **Hong Du:** funding acquisition. **Di Mei:** project administration. **AnQi Gu:** writing – review and editing. **BeiNa Hu:** writing – review and editing. **HaiQiang Jiang:** data curation.

## Ethics Statement

The authors have nothing to report.

## Conflicts of Interest

The authors declare no conflicts of interest.

## Data Availability

The authors confirm that the data supporting the findings of this study are available within the article.
